# Crosstalk of disulfidptosis-related subtypes identifying a prognostic signature to improve prognosis and immunotherapy responses of clear cell renal cell carcinoma patients

**DOI:** 10.1186/s12864-024-10307-0

**Published:** 2024-04-26

**Authors:** Lei Ren, Jinwen Liu, Qingyuan Lin, Tianyi He, Guankai Huang, Weifeng Wang, Xunhao Zhan, Yu He, Bin Huang, Xiaopeng Mao

**Affiliations:** 1 Department of Urology, The First Affiliated Hospital of Sun Yat-sen University, Sun Yat-sen University, Guangzhou, China; 2 Department of Urology, The Seventh Affiliated Hospital of Sun Yat-sen University, Sun Yat-sen University, Shenzhen, China; 3 Department of Breast Surgery, The First Hospital of China Medical University, Shenyang, China; 4 Department of Urology, Hui Ya Hospital of The First Affiliated Hospital of Sun Yat-sen University, Sun Yat-sen University, Huizhou, China

**Keywords:** Disulfidptosis, Clear cell renal cell carcinoma, Immunotherapy, Immune evasion, Tumor immune microenvironment

## Abstract

**Background:**

Disulfidptosis is a novel form of programmed cell death induced by high SLC7A11 expression under glucose starvation conditions, unlike other known forms of cell death. However, the roles of disulfidptosis in cancers have yet to be comprehensively well-studied, particularly in ccRCC.

**Methods:**

The expression profiles and somatic mutation of DGs from the TCGA database were investigated. Two DGs clusters were identified by unsupervised consensus clustering analysis, and a disulfidptosis-related prognostic signature (DR score) was constructed. Furthermore, the predictive capacity of the DR score in prognosis was validated by several clinical cohorts. We also developed a nomogram based on the DR score and clinical features. Then, we investigated the differences in the clinicopathological information, TMB, tumor immune landscapes, and biological characteristics between the high- and low-risk groups. We evaluated whether the DR score is a robust tool for predicting immunotherapy response by the TIDE algorithm, immune checkpoint genes, submap analysis, and CheckMate immunotherapy cohort.

**Results:**

We identified two DGs clusters with significant differences in prognosis, tumor immune landscapes, and clinical features. The DR score has been demonstrated as an independent risk factor by several clinical cohorts. The high-risk group patients had a more complicated tumor immune microenvironment and suffered from more tumor immune evasion in immunotherapy. Moreover, patients in the low-risk group had better prognosis and response to immunotherapy, particularly in anti-PD1 and anti-CTLA-4 inhibitors, which were verified in the CheckMate immunotherapy cohort.

**Conclusion:**

The DR score can accurately predict the prognosis and immunotherapy response and assist clinicians in providing a personalized treatment regime for ccRCC patients.

**Supplementary Information:**

The online version contains supplementary material available at 10.1186/s12864-024-10307-0.

## Introduction

Renal cell carcinoma (RCC) is the most prominent type of kidney malignancies, comprising almost 4% of all types of cancers and 2% of cancer-related fatalities globally [1], which can be broadly classified into clear cell renal cell carcinoma (ccRCC), chromophobe RCC, and papillary RCC [2]. ccRCC is the most widespread histological form, constituting approximately 75% of entire RCC samples [2, 3]. Over the past two decades, it has been widely acknowledged that the tumor stage with a TNM staging system has been recognized as one of the most significant prognostic factors of RCC in clinical practice guideline [4]. For patients with localized tumors (stage I-III), the standard treatment is partial or radical nephrectomy, which can yield a significantly more favorable prognosis [5]. However, 25-30% of RCC patient manifest metastases at the time of diagnosis (mccRCC), which indicates a poor prognosis with 5-year cancer-specific survival rate of 26.7% [5, 6]. Over the past few years, tyrosine kinase inhibitors (TKIs) and immune checkpoint inhibitors (ICIs) combination have been recommended as the first-line treatment for advanced RCC, including pembrolizumab plus axitinib, nivolumab plus cabozantinib, and pembrolizumab plus lenvatinib [7]. Moreover, the immunotherapy combinations present a great benefit in the survival outcomes compared to monotherapy [7, 8]. Because of the heterogeneous nature of tumor microenvironment (TME) in ccRCC, approximately 30% of mccRCC patients are unable to take advantage of from the immunotherapy combinations and ultimately develop resistance [7–9]. Numerous models, including the IMDC model and the UCLA Integrated Staging System (UISS) model, have demonstrated good prognostic potential, yet their capacity to forecast response to immunotherapy is still limited [8]. Consequently, the identification of a dependable predictive model for evaluating response to immunotherapy is indispensable to offer personalized treatment and improve the prognosis for ccRCC patients.

In a recent study, Liu et al. observed that the cystine uptake mediated by the overexpressed SLC7A11 can suppress ferroptosis under glucose starvation conditions; meanwhile, it also promotes a novel form of programmed cell death, termed disulfidptosis [10]. The disulfide stress induced by glucose starvation in SLC7A11-high cells can promote aberrant disulfide bonding of the actin cytoskeleton, triggering disulfidptosis. It was also found that glucose transporter inhibitors could induce cell death in SLC7A11-high cancer cells via disulfidptosis and inhibit SLC7A11-high tumor proliferation. In recent years, more and more studies have demonstrated that ccRCC is essentially a metabolic disease with genetic abnormalities involved in metabolic reprogramming, such as the tricarboxylic acid (TCA) cycle, glutamine or pentose phosphate (PPP) pathways [11–15]. Moreover, energy metabolism was abnormally regulated by the glycolysis [16–18], mitochondrial bioenergetics [19], and lipid metabolism [20], which may activate the oncogenic signaling pathways to promote ccRCC. In this review, Zheng et al. concluded that disulfidptosis may play an important role in cancer metabolic therapy and serve as a novel target of cancer treatment [21]. Therefore, targeting disulfidptosis may provide novel therapeutic strategies in cancer treatment.

In this study, we first undertook a thorough examination of the potential effects of the disulfidptosis-related genes (DGs) in the somatic mutations, prognosis, clinical features, and tumor microenvironment landscapes of ccRCC patients. Then, we developed a disulfidptosis-related prognostic signature (DR score) through the consensus clustering algorithm, LASSO, and multivariate Cox regression analysis. Our study revealed that the DR score was a pivotal independent prognostic factor and showed excellent predictive capacity of prognosis in ccRCC patients. We also found that patients with high DR scores had more complicated tumor microenvironment (TME) landscapes. In addition, patients with low DR scores respond better to immunotherapy. Finally, we verified the mRNA expression levels of disulfidptosis-related genes in RCC cell lines by qRT-PCR experiment.

**Fig. 1:**
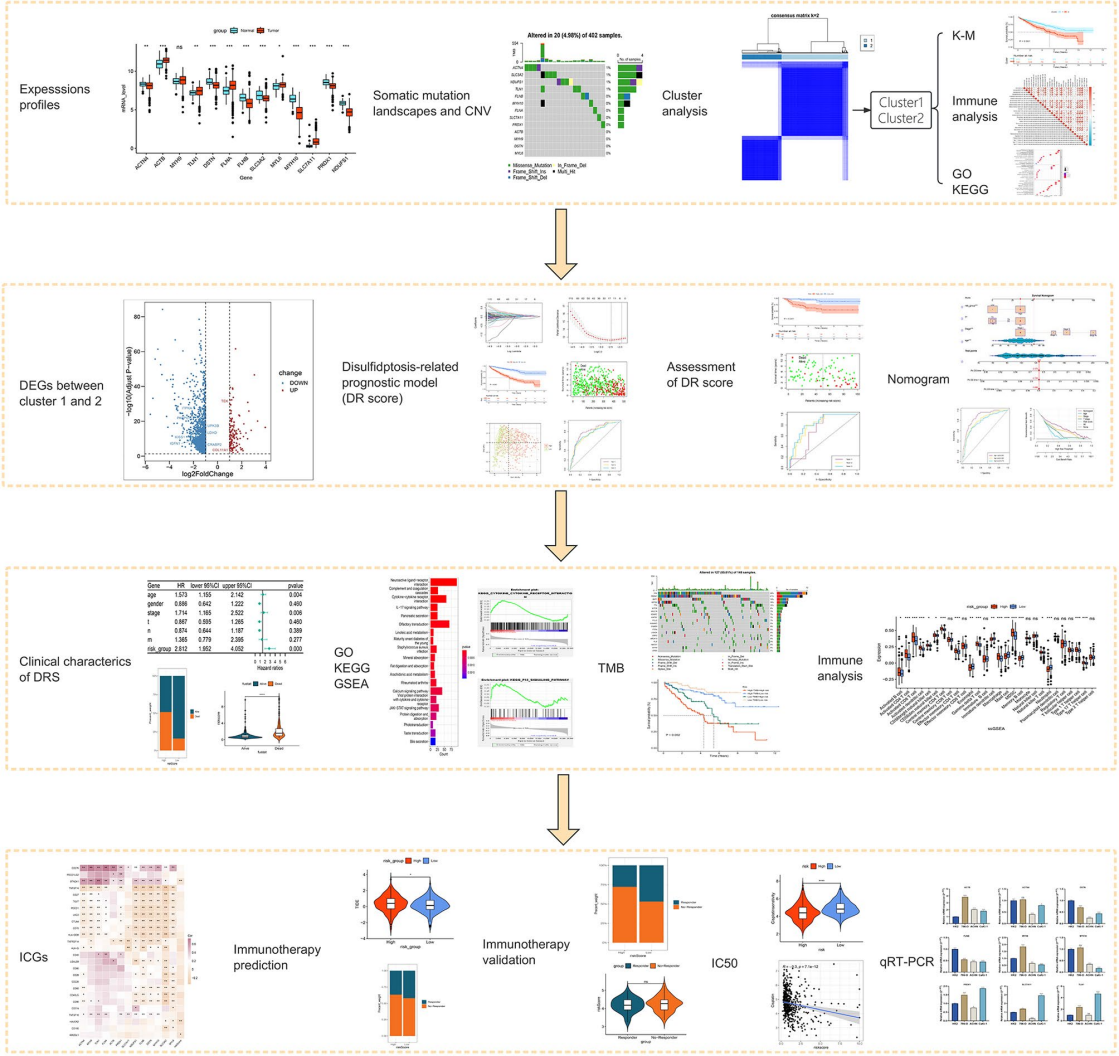
The flowchart of this study

## Methods and materials

### Sample data collection and procession

The original RNA-seq transcriptome data and clinicopathological information of the KIRC patients were downloaded from The Cancer Genome Atlas (TCGA) database (https://tcga-data.nci.nih.gov/tgca/ accessed October 2023) and the ArrayExpress database of the E-MTAB-1980 cohort (https://www.ebi.ac.uk/arrayexpress/) [22]. The RNA-seq data of the GSE40435 were obtained from the Gene Expression Omnibus (GEO) database (https://www.ncbi.nlm.nih.gov/geo/). The RNA-seq data and clinical information of the Braun and JAVELIN trial cohorts, including ccRCC patients who received immune checkpoint inhibitors (ICIs) treatments, were obtained from previous literature [23, 24]. We extracted RNA-seq data in the transcripts per million (TPM) format and count data from the TCGA database. The log transformation was applied to normalize the RNA-seq. To minimize the possibility of bias, we removed any patients from these cohorts who had inadequate clinicopathological data or overall survival time (OS)/progression-free survival (PFS) of less than 30 days. Ultimately, our study included 581 patients (509 ccRCC samples and 72 normal tissues) from the TCGA database, 202 patents (101 RCC patients and 101 normal tissues) from the GSE40435, 99 ccRCC patients from the E-MTAB-1980 cohort, 167 ccRCC patients from the Braun cohort (CheckMate 010, 025), and 221 RCC patients from the JAVELIN trial cohort. Due to differences in the treatment strategies, which may limit the applicability of the prognostic signature, we included four databases of various treatment strategies. The most ccRCC patients in the TCGA and E-MTAB-1980 cohorts underwent radical nephrectomy, and a few patients received neoadjuvant treatment and postoperative adjuvant therapy. The Braun cohort patients with advanced ccRCC received Nivolumab immunotherapy (anti-PD-1), and the JAVELIN trial patients received Avelumab + Axitinib immunotherapy (anti-PD-L1 + TKIs). The details of these cohorts in clinical information were summarized in Additional file 2: Table **S1**.

Due to the significance of suppressor genes of disulfidptosis, we selected SLC7A11 and SLC3A2 which were identified as the top two suppressor genes of disulfidptosis for further analysis. NDUFS1, an important gene of mitochondrial oxidative phosphorylation, was also identified as top synergistic hits, and its inactivation could induce disulfidptosis. Peroxiredoxin-1 (PRDX1), which is an important disulfide-bonded protein, plays a significant role in redox maintenance and is also included in the study. MYH9, MYH10, and MYL6 are all myosin heavy and light chains; these proteins, filamin-A and -B (FLNA/FLNB), actin (ACTB), and talin-1 (TLN1) were the top proteins under glucose starvation conditions with increased disulfide bonds. In the process of disulfidptosis, alpha-actnin-4 (ACTN4) and destrin (DSTN) were important proteins to form the actin cytoskeleton organization. Therefore, we selected these important protein genes in the disulfidptosis to conduct the further analysis (Additional file 3:Table **S2**) [10]. The protein-protein interaction networks (PPI) of 13 DGs were constructed by the online tool GeneMANIA (https://genemania.org/). Immunohistochemical (IHC) staining files of DGs of ccRCC tissues and normal samples were retrieved from the Human Protein Atlas database (https://www.proteinatlas.org/).

### Identification of disulfidptosis-related subtypes

Unsupervised consensus clustering analysis of 509 ccRCC patients from the TCGA database was performed based on the expression profiles of 13 DGs by the “ConsensusClusterPlus” R package with K-means inner loop algorithm [25]. When the clustering index k = 2, the linkage between the clusters is tenuous, and the association within the cluster is strong. Therefore, two disulfidptosis-related gene clusters (DGs clusters) were identified in the TCGA cohort. We also calculated the principal component analysis (PCA) scores based on 13 DGs of the two DGs clusters, and the “ggplot2” and “factoextra” R packages were used to plot the PCA chart. Kaplan–Meier (K-M) survival method was implemented to differentiate the OS between the two DGs clusters via the “survival” package in R.

Differentially expressed genes (DEGs) between two DGs clusters (cluster 1 vs. cluster 2) were identified by the “Deseq2” R package with the standards of p. adj. <0.05 and|log_2_Fold Change (FC)| >1 for the next analyses [26]. We identified 1383 DEGs between two DGs clusters, shown in the volcano plot (Additional file 1: Fig. **S2****A, Additional file 3: Table ****S2**). Based on the “clusterProfiler” and “enrichplot” R packages, we subsequently implemented GO and KEGG functional enrichment analyses to discover associated enriched signaling pathways.

### Immune analysis

Based on the expression profiles and the “estimate” package in R, the TME scores, including the immune, stromal, and estimate scores (immune scores + stromal scores), were calculated by the ESTIMATE algorithm. The immune cell levels were evaluated using CIBERSORT algorithm. Then, the single-sample gene set enrichment analysis (ssGSEA) algorithm was conducted to determine the subsets of immune infiltrating cell levels by the “CSVA” R package. All these results were plotted as violin or box plots by the “ggpubr” and “ggplot2” packages in R. The “corrplot” R package was used to plot the correlations between the infiltration levels of immune cell subsets.

### Development and validation of disulfidptosis-related prognostic signature

Firstly, the TCGA cohort was used as the training set, while the E-MTAB-1980, Braun, and JAVEKIN trial cohorts were used as the testing set. Drawing from the overall survival time in the training set, univariate Cox analysis was executed to ascertain the OS-related DEGs (OS-genes) (Additional file 3:Table **S2**). To order to minimize the potential of overfitting, we implemented LASSO Cox regression analysis in the training set with 1,000 cycles by the “survival” and “glmnet” packages in R. Ultimately, we discovered appropriate optimal OS-genes and established a disulfidptosis-related prognostic signature (DR score) by multivariate Cox regression analysis. The DR score (risk score) for each patient was calculated using the formula: risk score = $\sum _{i=1}^{n}(coefi\left(OS-genei\right)*\text{e}\text{x}\text{p}(OS-genei\left)\right)$. Where coefi is the regression coefficient derived by multivariate Cox regression, and exp is the normalized expression levels of each OS-genes. Based on the formula, the risk scores of all patients in these cohorts were obtained and normalized. Then, we divided all patients into the low-risk and high-risk groups using the median risk score as the cut-off [27]. K–M survival analysis was performed to compare the OS or PFS between the two risk groups using the “survival” R package. Besides, the time-dependent ROC curve was leveraged to measure the effectiveness of the DR score in forecasting prognosis of ccRCC patients with the “survminer”, “survival”, and “timeROC” R packages. Based on the clinical information and DR score subgroups, the heatmaps of the expression profiles of eight optimal OS-genes were plotted by the “pheatmap” R package.

### Clinical characteristic of disulfidptosis-related prognostic signature and nomogram

Univariate and multivariate Cox regression analyses were performed to determine whether the risk score was an independent OS prognostic indicator in ccRCC patients among clinicopathologic features in the TCGA, E-MTAB-1980, and Braun cohorts. The results were shown in the forest plots by the “forestplot” R package. A nomogram integrating the DR score and clinical characteristics (age, tumor stage, and T stage) was established to further augment the clinical implementation of the DR score by the “rms”, “foerign”, and “survival” R packages. The calibration curve and concordance index (C-index) were utilized to evaluate the prognostic performance of the nomogram. Decision curve analysis (DCA) was applied to evaluate the clinical benefits between the nomogram, risk score, and different clinical features by the “rmda” package in R.

### Tumor mutation burden analysis

We retrieved copy number variation (CNV) files and the somatic mutation data of ccRCC patients from the TCGA database. Based on the “maftools” R package, the mutations of 13 DGs were analyzed, and the CNV changes of these genes were also analyzed, shown in the bar plots. The locations of 13 DGs in chromosomes were obtained from the Ensembl (https://feb2104.archive.ensembl.org/). The “RCircos” package in R was utilized to plot the circle diagram of genes’ locations and CNV in chromosomes. Then, we calculated tumor mutation burden (TMB) scores of each patient and assessed the correlations between the TMB score and the DR score using the “maftools” package in R. According to the median value of TMB score, patients in the TCGA cohort were divided into the low- and high-TMB groups, and K-M survival analysis was used to compare the differences in the OS between two TMB groups. Additionally, we split all patients into four categories using the DR and TMB scores combination, and K-M survival analysis was also performed.

### Prediction and verification of immunotherapy responses

To assess immunotherapy outcomes and responses of ccRCC persons in the TIDE website (https://tide.dfci.harvard.edu/), we retrieved the tumor immune dysfunction and exclusion (TIDE) scoring, determined the TIDE, immune dysfunction, immune exclusion scores, as well as CD274 levels, and evaluated responses to immunotherapy. Then, we forecasted responses to anti-CTLA4 and anti-PD1 immunotherapy between two DR score groups through the R package “submap”. Additionally, we investigated underlying differences in immune checkpoint genes (ICGs) expression levels between two DR score groups. The underlying relationships between DR score, 13 DGs and ICGs expression levels were assessed, which were effective makers of immunotherapy responses. The correlation analysis p value between the ICGs and DR score, 13 DGs were adjusted by the Bonferroni multiple testing correction. In the Braun (CheckMate) cohort, patients with complete response (CR) and partial response (PR) were classified as responders to immunotherapy, whereas patients with progressive disease were classified as non-responders to immunotherapy. To investigate differences between responder and non-responder groups, the chi-square test was applied.

### Prediction of chemotherapy

The cancer-associated fibroblast (CAF), tumor drug resistance-related cell tumor-associated macrophage (TAM.M2), and myeloid-derived suppressor cell (MDSC) were calculated to assess the relationship between the risk score and chemotherapy responses. Based on the “oncoPredict” R package, we predicted the half-maximal inhibitory concentration (IC50) values of the widely recognized anti-RCC drugs for the low- and high-risk groups on the Genomics of Drug Sensitivity in Cancer (GDSC; https://www.cancerrxgene.org/) database. The correlations between the IC50 of chemotherapy drugs and the risk score were evaluated.

### Cell culture

Human ccRCC cancer cells ACHN, CAKI-1, and 786-O and human renal proximal convoluted tubule cell line HK-2 were purchased from Procell (Procell Life Science & Technology Co., Ltd). Cells were cultured in RPMI-1640 medium (Invitrogen) mixed with 10% FBS. The incubator was set in a water-saturated atmosphere with 5% CO_2_ at 37 °C.

### Quantitative real-time PCR (qRT-PCR)

The TRIzol Reagent (Invitrogen, USA) was applied to extract total cellular RNA according to the protocol. RNA was reverse transcribed to cDNA by the PrimeScript RT reagent kit (EZBioscience, China). EZBioscience 2 × SYBR Green qPCR Master Mix (EZBioscience, China) conducted the procedure. Primers for mRNAs were provided by TSINGKE (Beijing TSINGKE Biotech Co., Ltd., China) and shown in Additional file 5:Table **S4**. GAPDH was chosen as internal reference. Expression levels of mRNAs were measured as 2^−ΔΔCT^.

### Statistical analysis

R software (ver.4.3.1) was used to analyze data and visualize the results. Gene set enrichment analysis (GSEA) was conducted to analyze potentially enriched pathways of two risk groups using GSEA software (version 4.3.2, https://www.gsea-msigdb.org); meanwhile, p. adj. < 0.05 and simulated value = 1,000 were considered statistically significant. Spearman correlation analysis was utilized to ascertain the correlation coefficient among variables. Statistically significant differences between K-M survival curves was determined by the log-rank test [28]. Wilcox t-test was utilized to compare the differences of variables between the two groups. A p-value of less than 0.05 was determined to be the benchmark for statistical significance in the absence of any further explanation.

**Fig. 2:**
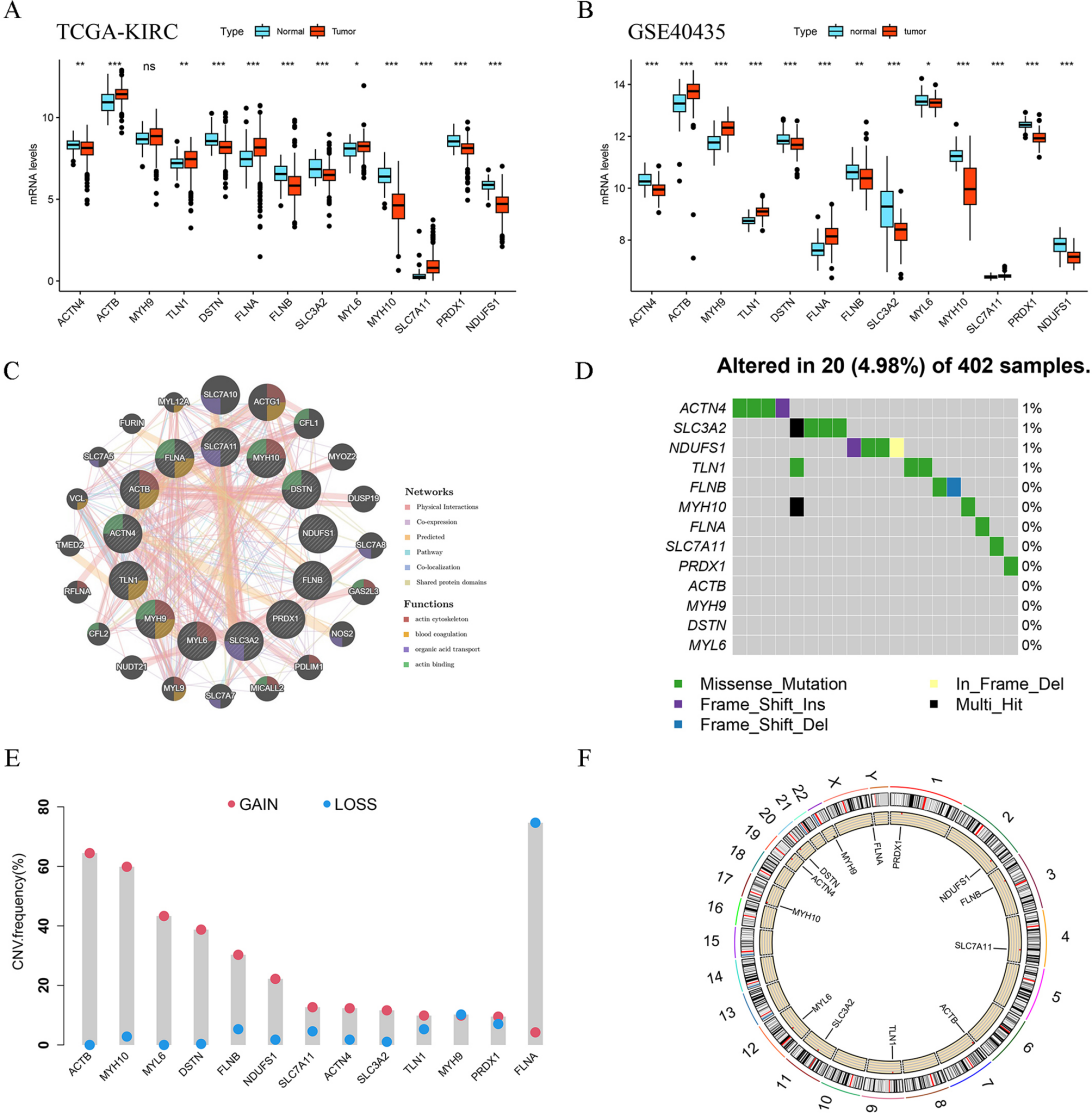
The expression profiles and mutational characteristics of disulfidptosis-related genes. **A**-**B** Expression profiles of 13 DGs between ccRCC tissues and normal samples in the TCGA and GSE40435 cohorts, respectively. **C** The PPI network of 13 DGs by GeneMANIA. **D** Somatic mutation of 13 DGs in ccRCC patients. **E** The frequency of copy number variations in 13 DGs. **F** The circus plot of CNV on chromosome location in 13 DGs. *P < 0.05, **P < 0.01, ***P < 0.001, ns: no significance

## Results

### Expression profiles and transcriptional mutation of disulfidptosis-related genes in ccRCC

The flowchart of our study is illustrated in Fig. 1.

First, we analyzed mRNA expression levels of 13 DGs between ccRCC and normal tissues in the TCGA and GSE40435 databases (Fig. 2A, B). Our study revealed considerable downregulation of ACTN4, DSTN, FLNB, SLC3A2, MYH10, PRDX1, and NDUFS1 in ccRCC tissues. Conversely, ACTB, MYH9, TLN1, FLNA, MYL6, and SLC7A11 were significantly overexpressed in ccRCC tissues. We also investigated the protein levels of these genes in ccRCC and normal tissues by IHC staining, which revealed the same results with the mRNA expression levels (Additional file 1: Fig. **S1****A**). These findings suggest that disulfidptosis may play a vital role in the development of ccRCC. Through the protein-protein interaction (PPI) network analyzed by the online tool GeneMANIA, we found that ACTB, MYH9, FLNA, and MYH10 were the hub-genes in this PPI network (Fig. 2C). Then, we evaluated the incidence rate of the somatic mutation and CNV alterations in 13 DGs in ccRCC patients. As shown in Fig. 2D, only 20 (4.98%) of 402 ccRCC patients had somatic mutation, and ACTN4, SLC3A2, NDUFS1, and TLN1 had a 1% incidence of missense mutation. The results of CNV alterations in 13 DGs were shown in Fig. 2E, which indicates that the CNVs of ACTB, MYH10, MYL6, DSTN, FLNB, and NDUFS1 were markedly increased, whereas the CNV of FLNA was significantly decreased. The location of the CNV in 13 DGs on chromosomes was displayed in a circle diagram (Fig. 2F).

**Fig. 3:**
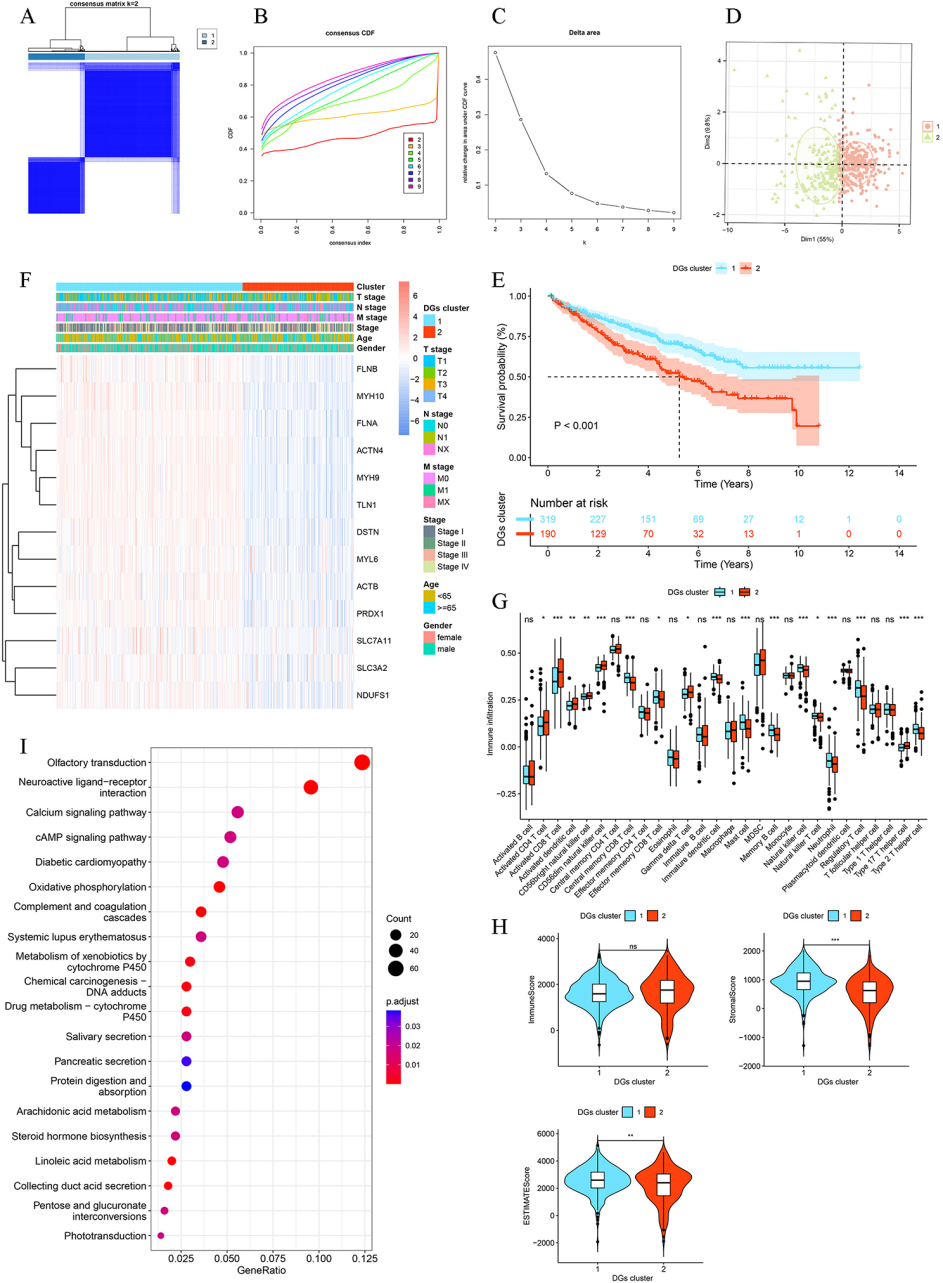
Identification of two DGs clusters and differences in prognosis, TME, and clinicopathological features. **A-C** All ccRCC patients from the TCGA cohort were divided into two DGs clusters by unsupervised consensus clustering analysis (k = 2). The Cumulative Distribution Function (CDF) curve for k = 2–9. Area under the CDF curve for k = 2–9. **D** The principal component analysis displays significant differences of two DGs clusters. **E** Kaplan–Meier survival curve of two DGs clusters. **F** The heatmap includes clinicopathological features and expression profiles of 13 DGs in the TCGA cohort. **G** The infiltration levels of 28 human immune cell subtypes between two DGs clusters. **H** The Immune, Stromal, and ESTIMATE scores of two DGs clusters. **I** KEGG analysis of DEGs in two DGs clusters. *P < 0.05, **P < 0.01, ***P < 0.001, ns: no significance

**Fig. 4:**
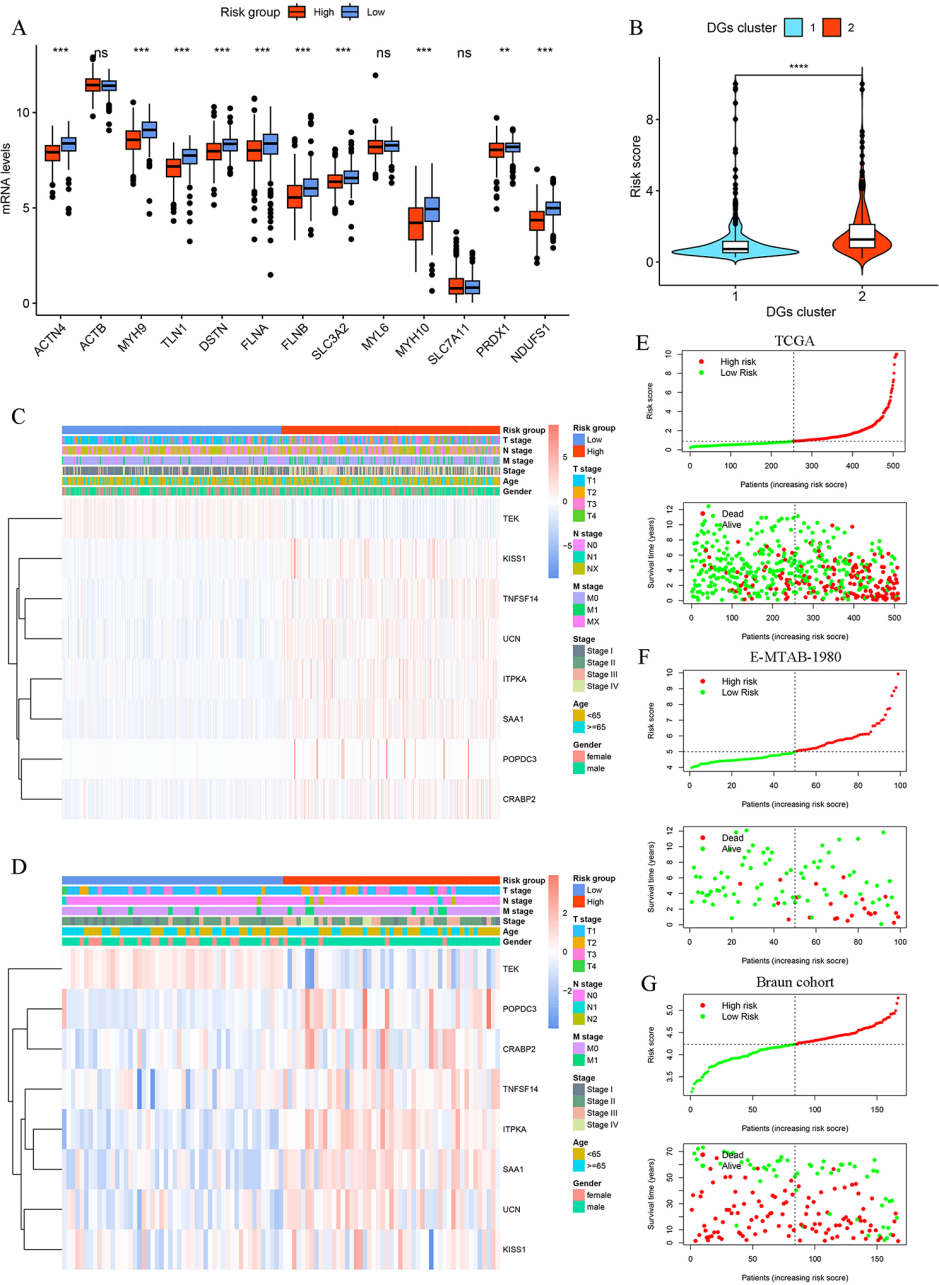
Development of the disulfidptosis-related prognostic signature (DR score). **A** The expression profiles of 13 DGs between the low- and high-risk group in the TCGA cohort. **B** Difference of DR scores between two DGs clusters. **C-D** The heatmap includes clinicopathological features and expression profiles of 8 OS-genes in the TCGA and E-MTAB-1980 cohort, respectively. **E-G** The distribution of DGs scores and survival status in the TCGA, E-MTAB-1980, Braun cohort, respectively. *P < 0.05, **P < 0.01, ***P < 0.001, ns: no significance

### Identification of disulfidptosis-related subtypes and tumor microenvironment analysis in ccRCC

To investigate the relationships among disulfidptosis regulators and ccRCC, we performed the consensus clustering algorithm analysis based on the expression profiles of 13 DGs. According to the lower slope of the Cumulative Distribution Function (CDF) curve, we divided all patients from the TCGA cohort into two clusters (Fig. 3A-C). As displayed in Fig. 3D, the result of PCA revealed the remarkable otherness of the distribution of two DGs clusters. The K-M curves showed that patients in the DGs cluster 2 had a worse prognosis than those in cluster 1 (Fig. 3E). Then, the gene expressions of DGs and clinicopathological features between two DGs clusters were also evaluated. As shown in Fig. 3F, the two DGs clusters presented a significant difference in DGs expression levels; based on the chi-squared test or Fisher’s test, we found that the tumor stage, T stage, and M stage showed significant difference (*p* < 0.05) between the two DGs clusters and the DGs cluster 2 had higher tumor stage, T stage, and M stage, which may account for the worse prognosis.

Based on the CIBERSORT, ssGSEA, and ESTIMATE algorithms, we examined the associations between TME and 13 DGs. We found that DGs cluster 1 had more macrophages and B cells naïve fractions in TME, while cluster 2 had more T cells CD8^+^ and NK cells fractions (Additional file 1: Fig. **S1****B, C**). As presented in Fig. 3G, higher levels of central memory CD8^+^ T cell, effector memory CD8^+^ T cell, mast cell, and regulatory T cell infiltrated in the DGs cluster 1, whereas cluster 2 had more infiltration of activated CD8^+^ T cell, activated CD4^+^ T cell, CD56dim natural killer cell, and type 17 T helper cell. Additional file 1: Fig. **S1****D** showed the correlations of the infiltration between 28 human immune cell subtypes. In addition, the results of ESTIMATE algorithm revealed that the DGs cluster 1 had a higher Stromal and ESTIMATE scores than cluster 2, indicating the more complicated TME in the DGs cluster 1 (Fig. 3H).

To investigate further the biological mechanisms behind apparent discrepancies between the two DGs clusters, we performed a functional enrichment analysis based on the DEGs between the two clusters. KEGG analysis revealed that these DEGs were mainly enriched in calcium signaling pathway, cAMP signaling pathway, oxidative phosphorylation, and complement and coagulation cascades (Fig. 3I). GO analysis indicated that DEGs exhibited significant enrichment mostly in humoral immune response (Additional file 1: Fig. **S1****E**).

### Establishment and verification of disulfidptosis-related prognostic signature

The TCGA cohort was regarded as the training set, while the E-MTAB-1980, Braun, and JAVELIN trial cohorts were regarded as the testing sets. Based on the OS-genes, LASSO, and multivariate Cox regression analysis (Additional file 1: Fig. **S2****B, C**), we established a disulfidptosis-related prognostic signature to improve prognosis and immunotherapy responses of ccRCC patients, which included eight prognosis-related genes. Then, we calculated the risk scores (DR score) of each patient with the equation below: DR Score = (-0.14807 * TEK) + (0.108541 * TNFSF14) + (0.266526 * POPDC3) + (0.153862 * ITPKA) + (0.121762 * CRABP2) + (0.228253 * UCN) + (0.225723 * KISS1) + (0.048607 * SAA1). On the basis of the median DR score threshold, patients in these cohorts were categorized into two groups: low-risk and high-risk.

**Fig. 5:**
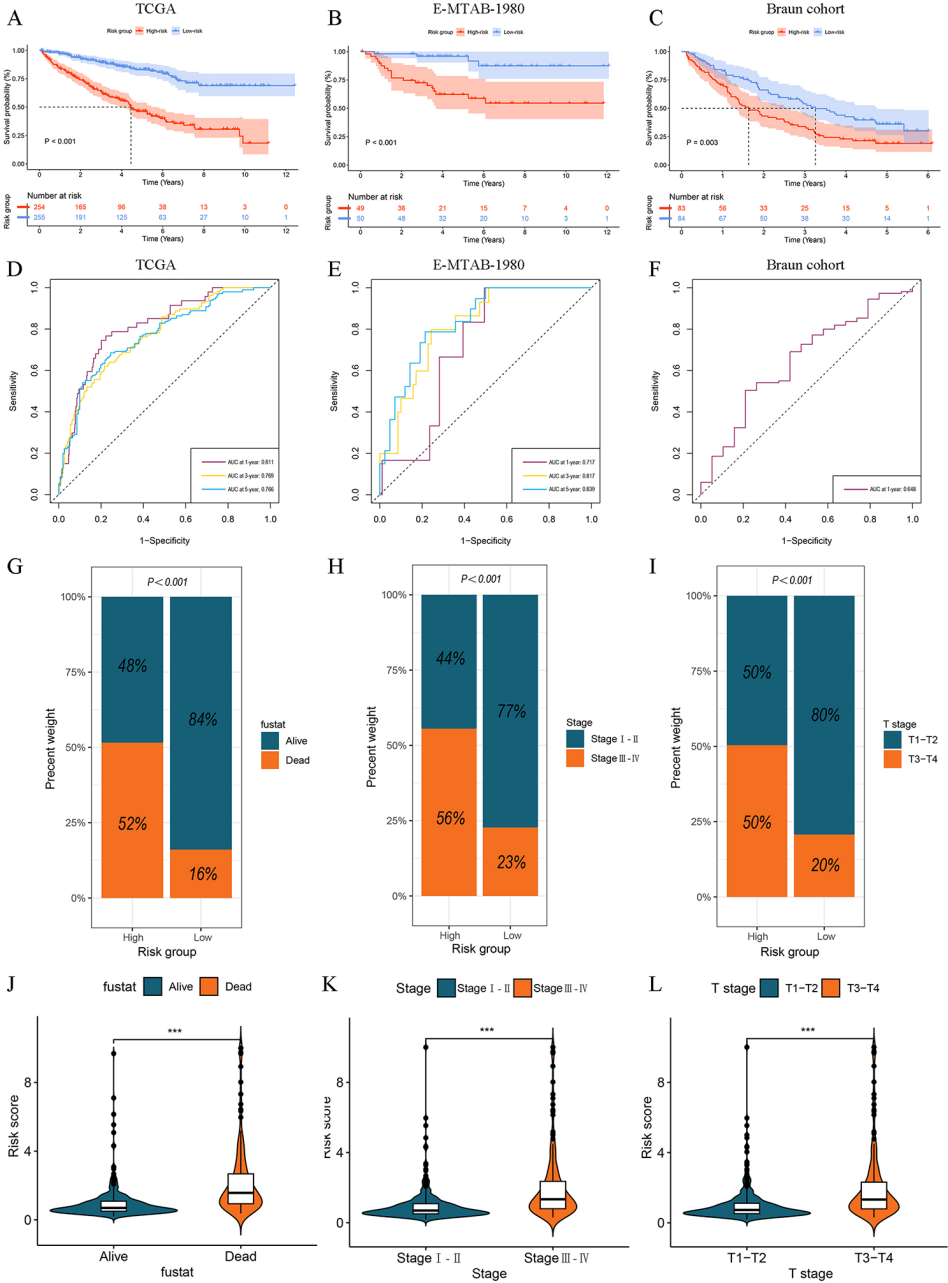
The relationships between prognosis or clinicopathological features and DR scores. **A-C** Kaplan–Meier survival curve of two DR score group in the TCGA, E-MTAB-1980, Braun cohort, respectively. **D-F** The time-ROC curves of DR scores in the TCGA, E-MTAB-1980, Braun cohort, respectively. **G-I** Distribution of survival status, tumor stage, and T stage between the two DR score group in the TCGA cohort. **J-L** Difference of DR scores when stratified by survival status, tumor stage, and T stage. *P < 0.05, **P < 0.01, ***P < 0.001, ns: no significance

We found that most DGs were significantly upregulated in the low-risk group (Fig. 4A, Additional file 1: Fig. **S2****D**). Then, we explored the relationship between the DR score and two DGs clusters. As displayed in Fig. 4B, the DGs cluster 2 had higher DGs scores than cluster 1. Then, the expression levels of eight prognosis-related genes and clinicopathological features between the two risk groups were also investigated in the TCGA and E-MTAB-1980 cohorts. We found that expression levels of prognosis-related genes and clinical features between two risk groups were significantly different (Fig. 4C, D). The comparison in the DG score distribution and survival status between two risk groups in the training and testing sets demonstrated that the DR score effectively differentiated ccRCC patients (Fig. 4E-G, Additional file 1: Fig. **S2****E**). The survival analysis of the training set showed that the high-risk group patients exhibited poorer prognosis than those in the low-risk group, which was consistent with the survival analysis results of the testing sets (Fig. 5A-C, Additional file 1: Fig. **S2****F**). The results of time-dependent ROC curve analysis demonstrated that the areas under the curve values (AUCs) of the DR score in predicting OS were all more than 0.710 for 1-, 3-, and 5-years in the TCGA and E-MATB-1980 cohorts (Fig. 5D, E). The AUCs of the Braun and JAVELIN trial cohorts also showed good prediction of OS (Fig. 5F, Additional file 1: Fig. **S2****G**). The aforementioned findings together demonstrated that the DR score exhibited a high level of predictive efficacy in relation to OS.

**Fig. 6:**
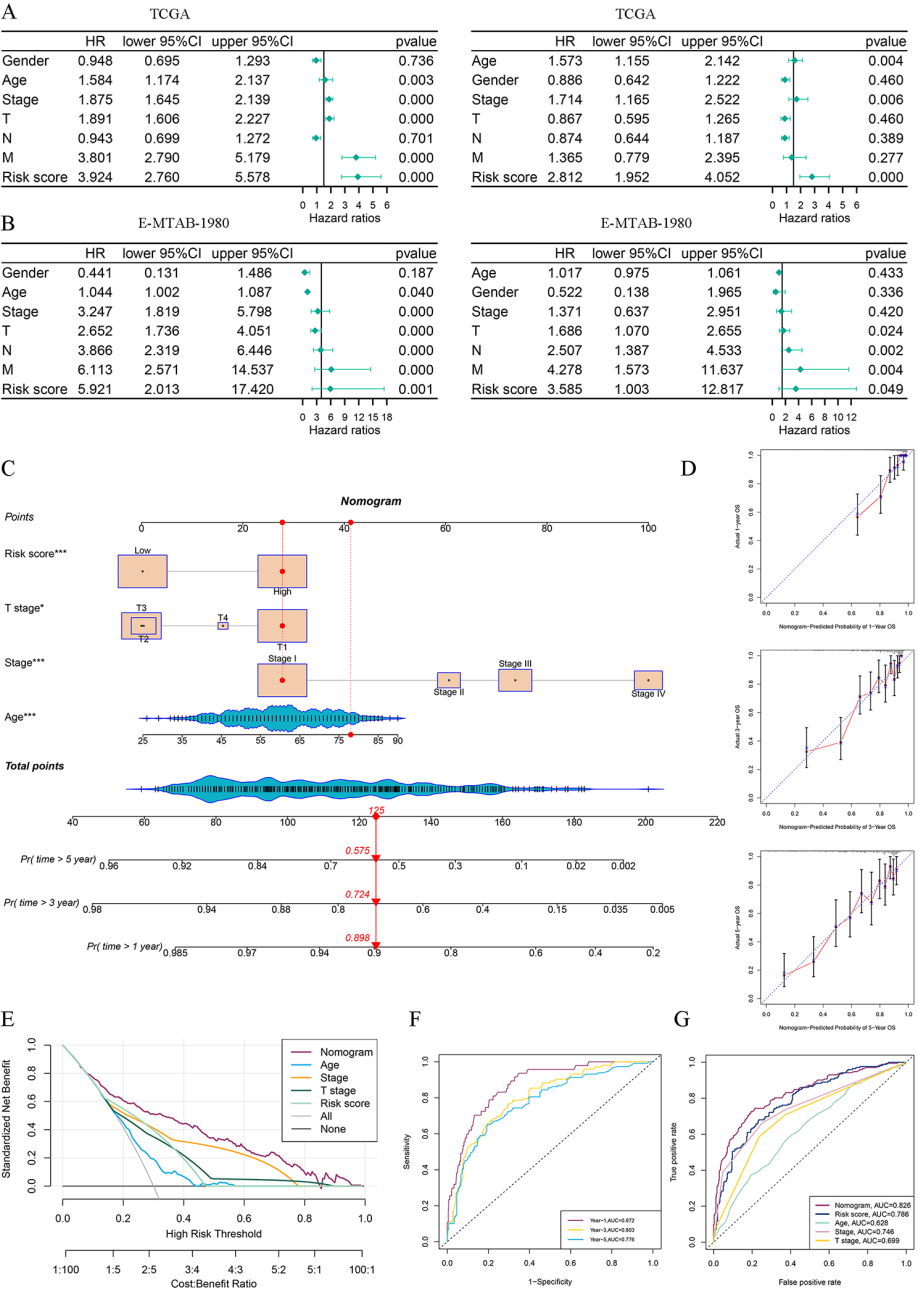
Construction and assessment of nomogram. **A-B** Univariate and multivariate Cox regression analyses of DR scores and clinicopathological features in the TCGA and E-MTAB-1980 cohort, respectively. **C** Nomogram integrated the DR score and clinical features. **D** The calibration curves displayed good uniformity between the predicted 1, 3, 5-years OS by the nomogram and the actual OS. **E** Decision curve analysis of nomogram, DR score, and clinical features. **F** The time-ROC curves of nomogram. **G** Multivariate ROC curves analysis of nomogram, DR score, and clinicopathological features

### Clinical significance of disulfidptosis-related prognostic signature and nomogram

To investigate the clinical significance of the DR scores, we compared the clinicopathological features (including survival state, tumor stage, and T stage) between two risk groups and the DR scores between different subgroups stratified by clinicopathologic features. As portrayed in Fig. 5G-L, it was observed that the high-risk group patients had a significant correlation with unfavorable OS, higher tumor stage and T stage, which revealed that from the clinical standpoint, the higher DR score indicates a higher degree of tumor malignancy. In order to provide further evidence, we conducted the same investigations on the E-MATB-1980 cohort and achieved the same findings, so validating our conclusion (Additional file 1: Fig. **S3****A, B**). Furthermore, univariate and multivariate Cox regression analyses proved that the DR score is an independent risk factor among other clinicopathological features (Fig. 6A, B, Additional file 1: Fig. **S3****C**). A nomogram was developed by incorporating tumor stage, age, T stage, and the DR score (Fig. 6C). The calibration curves for 1, 3, and 5-years OS rates demonstrated excellent performance of the nomogram in prognostic prediction (Fig. 6D). The C-index of the nomogram was 0.779 (95% CI 0.741–0.811, *p* = 0.0025). The findings from DCA indicated that the nomogram presented a greater net benefit in forecasting the prognosis compared to the DR score and other clinicopathological features (Fig. 6E). The AUCs of time-ROC about the nomogram were for 0.872, 0.803, and 0.776 for 1-, 3- and 5-years OS, respectively (Fig. 6F). The multivariate ROC curve revealed that our nomogram had highest AUC value with 0.826, while the DR score with 0.786 (Fig. 6G). These findings indicated that our nomogram could further enhance the accuracy of prognostic prediction for the DR score.

### Functional Enrichment Analysis and Gene set enrichment analysis of the DR score

**Fig. 7:**
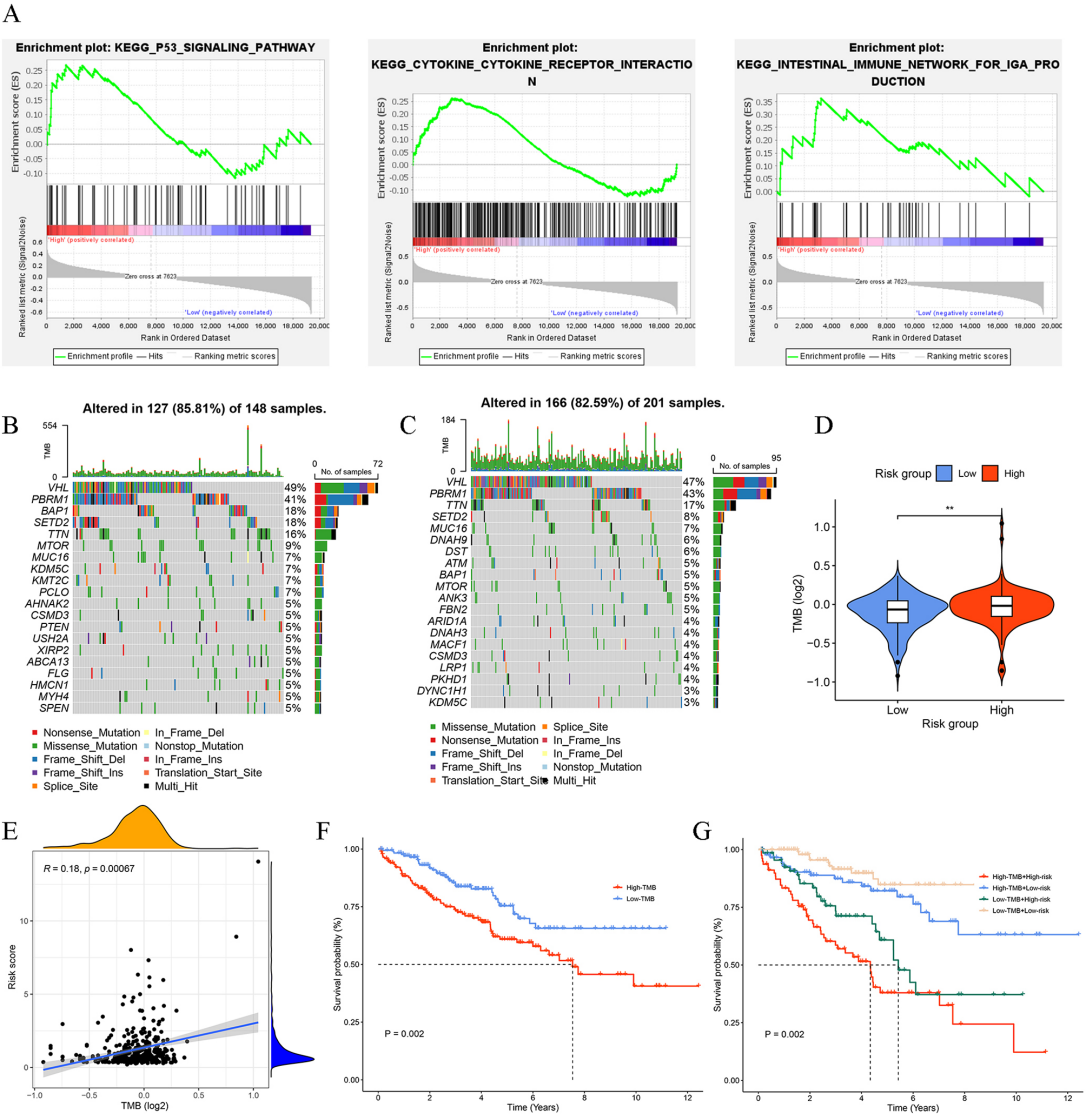
Analysis of tumor mutational landscapes based on DR score. **A** GSEA analysis showed pathways significantly enriched in the high-risk group. **B** Waterfall plots of mutation characteristics in the high-risk group. **C** Waterfall plots of mutation characteristics in the low-risk group. **D** Difference in TMB between the two risk groups. **E** Spearman correlation analysis of DR score and TMB. **F** Kaplan–Meier survival curve between the high- and low-TMB groups. **G** Kaplan-Meier survival curve of the OS stratified by both TMB and DR score. *P < 0.05, **P < 0.01, ***P < 0.001, ns: no significance

Based on the “Deseq2” R package with the standards of p. adj. <0.05 and|log_2_Fold Change (FC)| >1, we identified 2173 differentially expressed genes (DEGs) between two risk groups. In order to delve further the molecular processes behind the observed disparity of the two risk groups, GO, KEGG, and GSEA analyses were conducted. The results of functional enrichment analyses indicated that DEGs were primarily enriched in humoral immune response, calcium signaling pathway, cytokine-cytokine receptor interaction, IL-17 signaling pathway, and JAK-STAT signaling pathway (Additional file 1: Fig. **S3****D, E**), which were associated with immune-related pathways. Then, the GSEA analysis demonstrated that P53 signaling pathway, cytokine-cytokine receptor interaction, and intestinal immune network for IGA production were mainly enriched in the high-risk group (Fig. 7A), whereas the low-risk group exhibited significant enrichment of renal cell carcinoma, mTOR signaling pathway, and fatty acid metabolism (Additional file 1: Fig. **S3****F**). It is reasonable to conclude that the DR score had a prominent relationship to immunity and tumorigenesis.

### Tumor mutation burden and survival analysis

In light of the genomic features in the tumorigenesis, development and treatment, we analyzed the somatic mutation data of the two risk groups based on DR scores. VHL and PBRM1 were the two most mutated genes in two risk groups (Fig. 7B, C), which was the same as in previous studies [23]. We quantified the somatic mutation as the TMB score and discovered that the high-risk group patients possessed higher TMB scores than the low-risk group patients (Fig. 7D). Spearman correlation analysis revealed a positive correlation between the DR score and TMB score (Fig. 7E). Based on the median TMB score, we divided patients into the high-TMB and low-TMB groups, and the survival analysis showed that patients in the high-TMB group had a worse prognosis than the low-TMB group (Fig. 7F). Furthermore, the DR scores and TMB scores were combined to forecast prognosis. It was observed that the high-risk + high-TMB group patients had the worst prognosis, whereas the low-risk + low-TMB group patients possessed the best prognosis (Fig. 7G). All these results revealed that the TMB scores and DR scores were significantly associated with the prognosis.

### Tumor immune microenvironment analysis

**Fig. 8:**
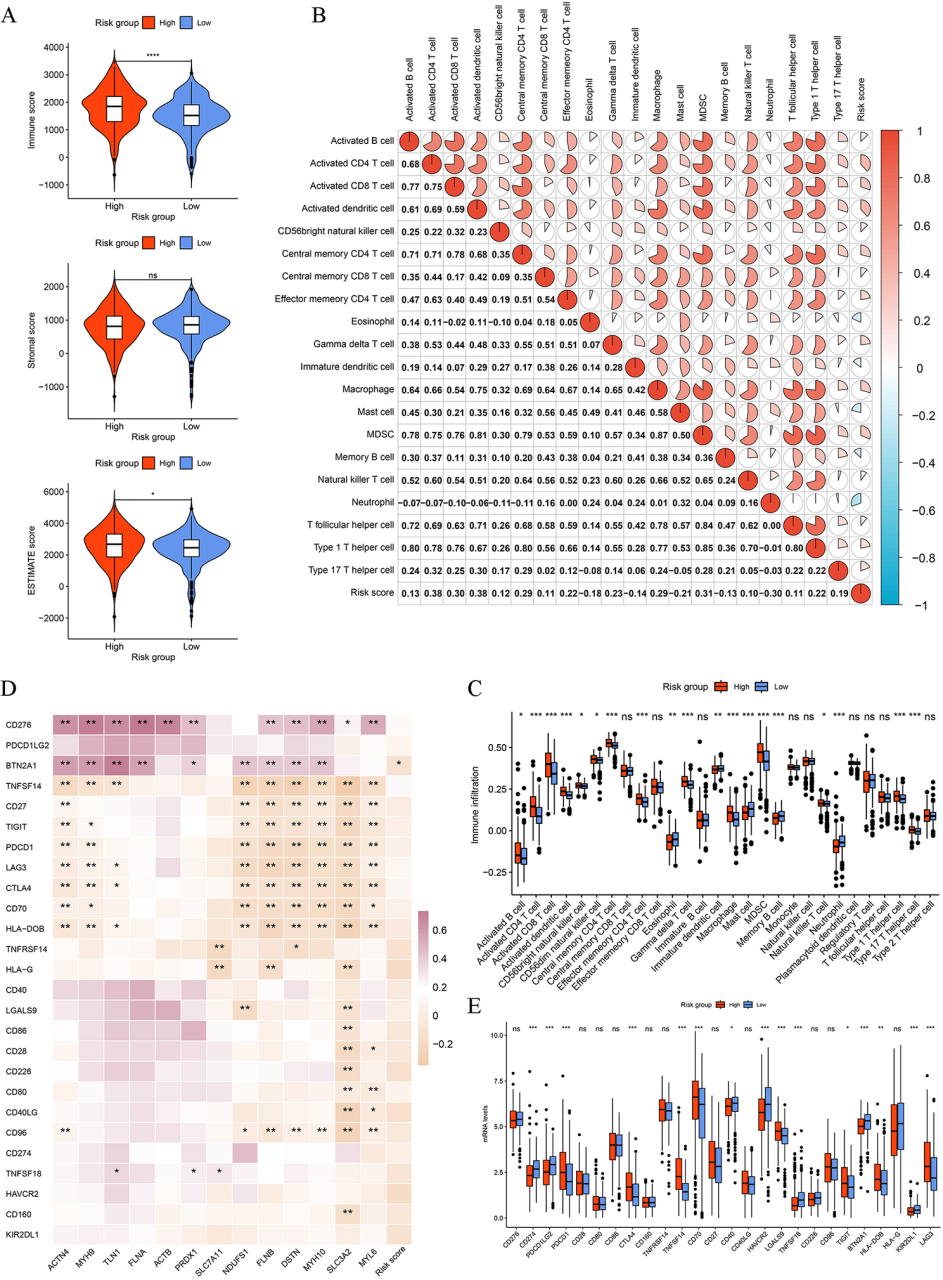
Tumor immune microenvironment analysis between two risk groups. **A** The Immune, Stromal, and ESTIMATE scores of two risk groups. **B** Correlation analysis of DR score with 28 immune cell subtypes infiltration levels. **C** The infiltration levels of 28 human immune cell subtypes between two risk groups. **D** Correlation analysis of DR score and expression levels of 13 DGs with immune checkpoint genes. **E** Expression levels of immune checkpoint genes between two risk groups. *P < 0.05, **P < 0.01, ***P < 0.001, ns: no significance

TME, comprising endothelial cells, fibroblasts, innate and adaptive immune cells, as well as non-cellular components, has generated increasing attention from research as an advanced biomarker in predicting responses to cancer immunotherapy [29, 30]. Firstly, we found that the high-risk group patients had higher Immune and ESTIMATE scores than those in the low-risk group (Fig. 8A). The results of ssGSEA indicated that patients with a high DR score showed higher levels of immune cell infiltration, consistent with functional enrichment analyses and GSEA analysis (Fig. 8B, C). The differences of 22 immune cell fraction between the two risk groups suggested that the high-risk group was infiltrated by a higher portion of plasma cells, CD8^+^ T cells, T cells regulated (Tregs), T cells follicular helper, and Macrophages M0, while a lower portion of CD4^+^ T cells memory resting, B cells naïve, monocytes, mats cells resting, dendritic cells resting, and Macrophages M2 (Additional file 1: Fig. **S4****A**). The correlation analysis showed that the DR score was positively correlated with dendritic cells resting, plasma cells, T cells follicular helper, CD8^+^ T cells, Tregs, and macrophages M0, whereas it was negatively correlated with mats cells resting, monocytes, T cells CD4^+^ memory resting, Macrophages M2 (Additional file 1: Fig. **S4****B, C**).

### Prediction of immunotherapy and chemotherapy responses

**Fig. 9:**
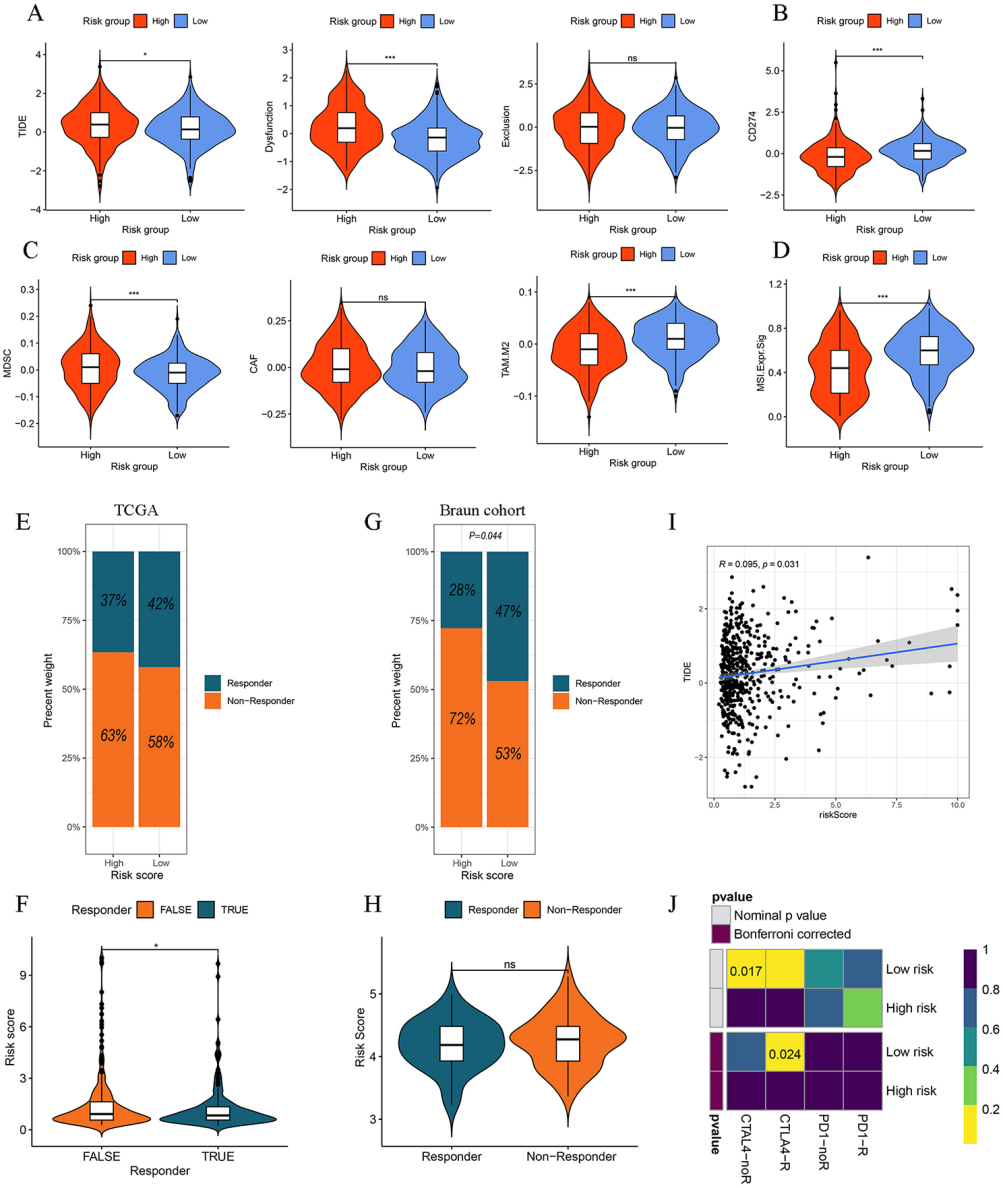
Predictive values of DR score in immunotherapy response. **A** TIDE, dysfunction, exclusion scores of two risk groups. **B** CD274 (PD1) levels calculated by the TIDE algorithm of two risk groups. **C** MDSC, CAF, and TAM. M2 scores of two risk groups. **D** MSI expression signature of two risk groups. **E** Distribution of responders and no-responders predicted by the TIDE algorithm bewteen two risk groups in the TCGA cohort. **F** Distribution of DR score between responders and no-responders predicted by the TIDE algorithm in the TCGA cohort. **G** Distribution of responders and no-responders between two risk groups in the Braun cohort (CheckMate). **H** Distribution of DR score between responders and no-responders between two risk groups in the Braun cohort (CheckMate). **I** Correlation analysis of the TIDE score and DR score. **J** Prediction of the response to anti-PD1 and anti-CTLA4 inhibitors in two risk groups by the submap algorithm. *P < 0.05, **P < 0.01, ***P < 0.001, ns: no significance

**Fig. 10:**
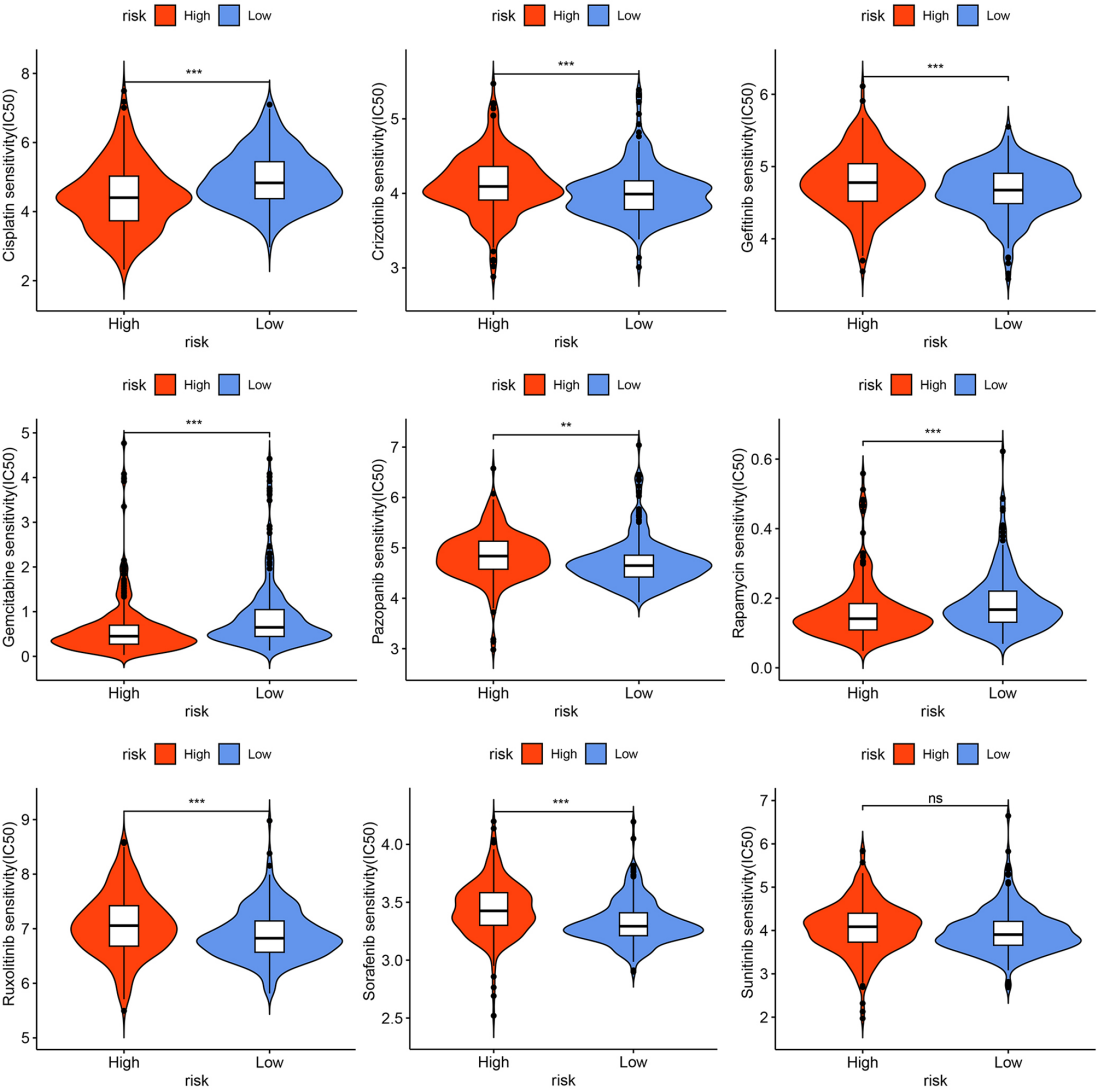
Prediction of sensitivity to chemotherapeutic drugs between the low- and high-risk groups. *P < 0.05, **P < 0.01, ***P < 0.001, ns: no significance

Immune checkpoint inhibitors (ICIs) have become an important component of the first-line treatment of advanced ccRCC [31]. Nevertheless, due to the heterogeneity of the cancer and TME, there is still a subset of patients who respond poorly to ICTs. Therefore, it is necessary to confirm which type of patients are suitable for ICIs. Firstly, we analyzed the expression levels of the common immune checkpoint genes between the different risk groups and found that patients with high DR scores had higher expression levels of PDCD1, CTLA4, TNFSF14, CD70, TIGIT, and LAG3, while patients with low DR score had higher expression levels of CD274, PDCD1LG2, HAVCR2, HLA-G, and KIR2DL1 (Fig. 8E). We also found that the 13 DGs and DR scores were significantly associated with the common immune checkpoint genes (Fig. 8D). Then, we also investigated the immunotherapy response between the two risk groups by the TIDE algorithm. As displayed in Fig. 9A **and I**, patients in the high-risk group had higher TIDE and dysfunction scores. It was also found that patients with low DR scores had higher expression levels of CD274 predicted by the TIDE algorithm (Fig. 9B), consistent with the actual level. We also found that patients who responded to immunotherapy showed lower DR scores than patients who had non-responder to immunotherapy (Fig. 9E, F). In the CheckMate immunotherapy cohort, patients in the low-risk group showed better responses to immunotherapy than those in the high-risk group (the chi-square test, *P* = 0.044), and patients who responded to immunotherapy had lower DR scores (Fig. 9G, H). Additionally, it was discovered that the low-risk group patients may be more likely to reap benefits of anti-CTLA-4 inhibitors (Fig. 9J, Bonferroni corrected *p* = 0.024). These results indicate that patients with low DR scores respond better to immunotherapy. We also found that patients in the low-risk group had higher levels of tumor-associated macrophages M2 (TAM M2) and microsatellite instability (MSI), while the high-risk group patients possessed elevated levels myeloid-derived suppressor cells (MDSCs) (Fig. 9C, D). Subsequently, responses to chemotherapeutic drugs in two risk groups were evaluated by the determination of IC50. The findings of the Spearman correlation analysis showed that the IC50 values of cisplatin, gemcitabine, and rapamycin were negatively correlated with the DR score, indicating that patients with high DR scores were sensitive to cisplatin, gemcitabine, and rapamycin (Fig. 10, Additional file 1: Fig. **S5**). Moreover, the IC50 values of crizotinib, gefitinib, pazopanib, sorafenib, and sunitinib were positively correlated with the DR score, revealing that patients with low DR scores were sensitive to crizotinib, gefitinib, pazopanib, and sorafenib (Fig. 10, Additional file 1: Fig. **S5**).

#### Expression levels of disulfidptosis-related genes in three ccRCC cell lines

**Fig. 11:**
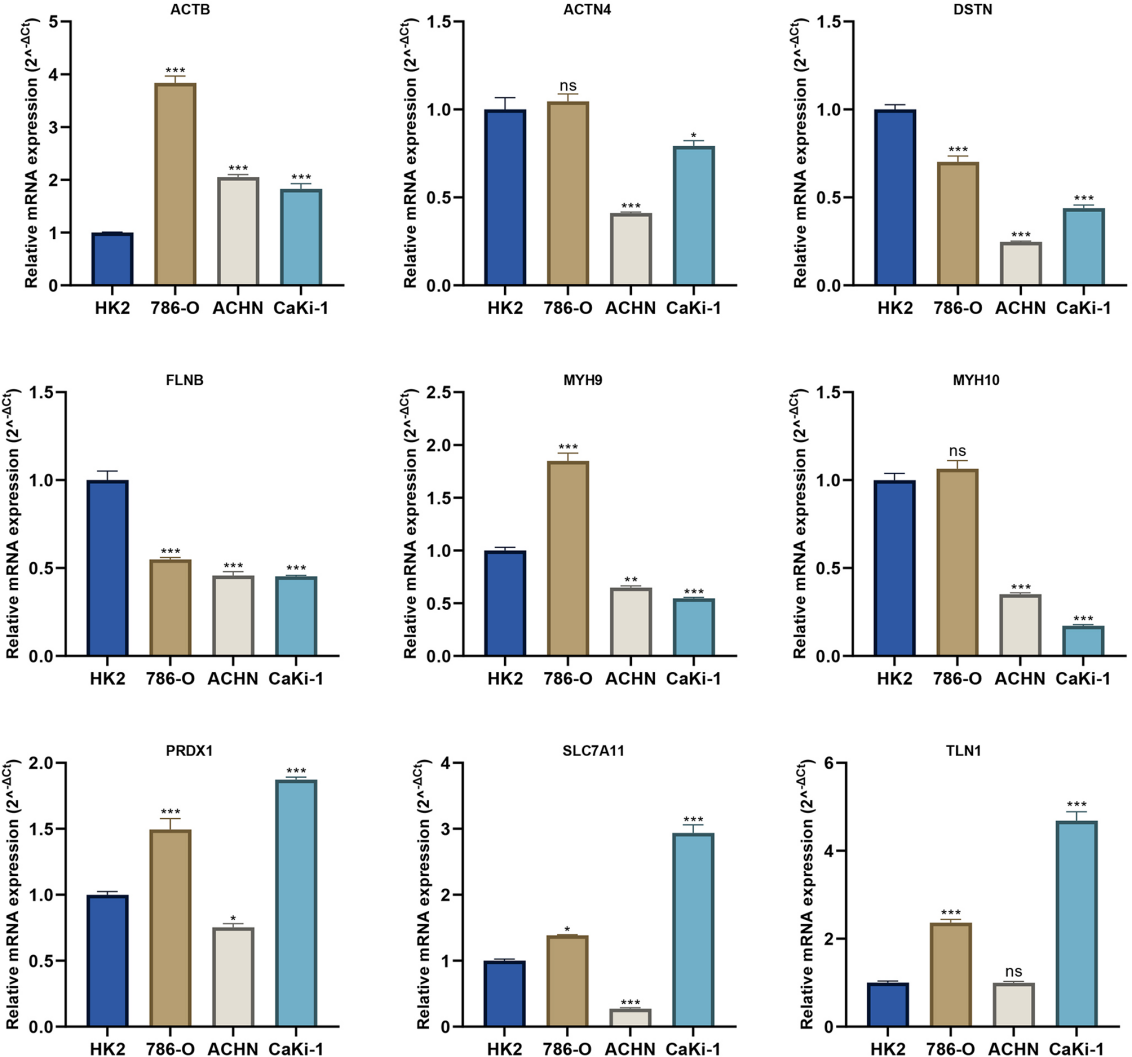
The mRNA levels of DGs (ACTB, ACTN4, DSTN, FLNB, MYH9, MYH10, PRDX1, SLC7A11, and TLN1) in three ccRCC cell lines by qRT-PCR. *P < 0.05, **P < 0.01, ***P < 0.001, ns: no significance

We also investigated the mRNA expression profiles of 13 disulfidptosis-related genes by qRT-PCR in three ccRCC cell lines (786-O, ACHN, and CaKi-1). As displayed in Fig. 11 and Additional file 1: S6, we found that mRNA levels of ACTB, PRDX1, SLC7A11, MYL6, SLC3A2, PRDX1, and TLN1 were significantly upregulated in ccRCC cell lines. It was also shown that mRNA levels of ACTN4, DSTN, FLNB, FLNA, MYH9, and MYH10 were downregulated in ccRCC cell lines. These mRNA levels of DGs by qRT-PCR were consistent with our bioinformatics analyses from the TCGA and GEO databases.

## Discussion

ccRCC is a highly heterogeneous and historically immunogenic tumor, with a notably infiltrations of immune cells compared to other solid tumors, especially T cells [9, 32–34]. Previous studies revealed that the activation of specific metabolic pathways plays an important role in regulating immunoflogosis in the ccRCC TME [35, 36]. Due to these characteristics of TME, the treatment regimens of ICIs, TKIs, and the combination of both developed rapidly and have been approved for the standard treatments in mccRCC patients. Although immunotherapy combinations can give a great benefit of survival in responders, the resistance will occur in most ccRCC patients, mainly resulting from the high heterogeneity of TME [32, 37–41]. Therefore, identifying accurate biomarkers for predicting responses to the immunotherapy of RCC patients is essential for clinicians to administer personalized therapies and improve prognosis of RCC patients.

The cystine transporter solute carrier family 7 member 11 (SLC7A11) plays a vital role in the glutathione (GSH) synthesis to suppress reactive oxygen species (ROS) and ferroptosis [42, 43]. Many studies revealed that the overexpression of SLC7A11 occurs in various types of malignancies, promotes the development and proliferation of them, and confers a survival advantage to cancer cells [44]. We also found that SLC7A11 is notably increased in ccRCC tissues in comparison to normal tissues. Moreover, the SLC7A11-high cancer cells are particularly susceptible to disulfidptosis under glucose starvation conditions; however, these cells are more likely resistant to apoptosis- and ferroptosis-inducing therapies. In addition, emerging evidences suggest that disulfidptosis can regulate the immune cells infiltration and immunoflogosis in glioma and colorectal cancer [45, 46]. Therefore, elucidating the molecular mechanism of disulfidptosis in ccRCC could provide novel perspectives into the innovative cancer treatment approaches aimed at targeting disulfidptosis.

In our present study, we initially investigated the genetic levels, the somatic mutation, and CNV alterations of DGs. It was found that most of the DGs were dysregulated in ccRCC tissues, especially SLC7A11, and only 4.98% of patients had somatic mutations of DGs. Based on the consensus clustering analysis of the mRNA expression profiles of 13 DGs, we developed two distinct DGs clusters (cluster 1 and cluster 2) of ccRCC patients from the TCGA cohort. In contrast to patients belonging to DGs cluster 2, the DGs cluster 1 patients had a more favorable prognosis and showed more complicated TME. The KEGG and GO analysis revealed that DEGs between two DGs clusters were mainly enriched in the cancer-related pathways, indicating a potential correlation between DGs and the progression and proliferation in ccRCC. Then, we identified 256 OS-genes among DEGs and established a disulfidptosis-related prognostic signature (DR scores) based on 8 OS-genes in the training set (TCGA cohort). The E-MTAB-1980, Braun, and JAVELIN trial cohorts were used as the testing sets. We found that patients in the DGs cluster 2 had higher DR scores than those in the cluster 1. The K-M curves revealed that the high-risk group patients possessed a more unfavorable prognosis compared to patients belonging to the low-risk group, which was demonstrated by both training and testing sets. To verify the applicability of our prognostic signature, we included these four cohorts with various treatment strategies. The TCGA cohorts and E-MTAB-1980 cohorts had similar treatment strategies; and most patients underwent radical nephrectomy. The Braun and JAVELIN trial cohorts aimed to evaluate the immunotherapy in ccRCC, which were entirely different from the training set. Moreover, our DR scores exhibited a strong predictive ability for OS across all cohorts, which demonstrated that the DR scores had very broad clinical applicability in predicting the prognosis in ccRCC patents with various different treatment strategies. In addition, it was also found that the DR score is an independent risk factor among other clinicopathological features in both training and testing sets. Nomograms are applied diffusely as statistical prognostic models to assist clinicians more easily understand the prognosis of tumor patients and provide compressive treatments [47, 48]. We also constructed a nomogram integrating the DR score and vital clinicopathological information (tumor stage, age and T stage), which has been proven to have a greater net benefit in forecasting the prognosis and improve the predictive accuracy of the DR score in OS with AUC 0.826. The most commonly used tumor stage system of RCC in clinical practice is the AJCC TNM system, which has been demonstrated as a prognostic factor in predicting outcomes for RCC patients [49]. Moreover, the tumor stage is significantly correlated with the grade malignancy and survival time of RCC [5, 50]. Therefore, between two risk groups, we compared some vital clinicopathological features (including survival state, tumor stage, and T stage) and found that patients in the high-risk group possessed worse outcomes, higher tumor stages, and higher T stages, which revealed that the high DR score represents the higher grade malignancy and more aggressive nature from the clinical point of view. GSEA results further showed a significant association between the DR score and tumor-related pathways and immunity.

Based on the somatic mutation data of ccRCC patients from the TCGA database, we found that VHL and PBRM1 were the top two mutated genes in the low- and high-risk groups, consistent with previous studies [23, 51]. We also found that patients in the high-risk group had higher TMB scores, and the TMB score is positively correlated with the DR score. Furthermore, patients with high TMB scores showed more unfavorable outcomes. It is well-known that TMB is an emerging predictive biomarker of response to immunotherapy in many solid tumors because of neoantigens generated by the somatic mutation genes in cancer cells to strengthen the immune reaction against tumors [8, 52–54]. In contrast to many other solid tumors, ccRCC has a solely moderate TMB [55]; although several studies investigate the TMB score as a predictive biomarker of response to immunotherapy in RCC, no definitive conclusion has been confirmed [23, 56].

In the last two decades, tumor microenvironment (TME) not only plays critical roles in ccRCC carcinogenesis, development, and invasion [32, 57], but it is also able to predict the immunotherapy response in many solid tumors, especially in ccRCC [9, 30, 37]. Therefore, we investigated the differences in TME between the two DR score groups. It was revealed that patients in the high-risk group possessed significantly elevated levels of the ESTIMATE and Immune scores, which indicated that this group has a more complicated tumor immune microenvironment. The infiltration of myeloid-derived suppressor cells (MDSCs), playing a suppressive role in immune responses and activating tumor immune escape [58], showed significantly increased levels in the high-risk group, including macrophages and immature dendritic cell. Moreover, the MDSC score estimated by the TIDE algorithm was also higher in the high-risk group. The other immunosuppressive cells, including CD8^+^ T cells, Tregs, T helper cells, and CD4^+^ T cells, were all significantly infiltrated higher in the high-risk group. Previous studies revealed that a higher proportion of CD8^+^ T cells and immunosuppressive macrophages M2 were infiltrated in the tissues of ccRCC compared to other cancers, and there exists a positive correlation between elevated infiltration levels of CD8^+^ T cells and unfavorable prognosis [30, 57]. Tregs, as one of the most important parts of immunosuppressive cells, can inhibit tumor-specific immune responses with the co-inhibitory molecules such as CTLA-4, TIGIT, LAG3, and CD28, and promote immune evasion, thus facilitating tumor cell proliferation [59–61]. The above co-inhibitory molecules of Tregs, also as the ICGs, were notably upregulated in the high-risk group patients. The PD-L1 (also known as CD274) expressed by tumor cells plays a crucial role in the mediation of immunosuppression [62], and its expression level in tumor cells has a potential relationship to objective response of immunotherapy of ccRCC, such as anti-PD1 inhibitors [63]. Surprisingly, we found that patients in the low-risk group had significantly higher expression levels of PD-L1 (CD274) than those in the high-risk group, and CD274 level calculated by the TIDE algorithm also demonstrated the results. In conclusion, these findings convincingly revealed that the high-risk group presents an immunosuppressive tumor microenvironment and suffers more tumor immune evasion in immunotherapy. The TIDE scores algorithm is widely applied in the prediction of immunotherapy response, especially in anti-PD1 inhibitors, which includes two classical mechanisms of tumor immune evasion: the induction of T cell dysfunction in tumor with high infiltration of CTL and the exclusion of T cell infiltration with low CTL [64]. Interestingly, our study revealed that the high-risk group patients showed higher TIDE scores and dysfunction scores, and patients who responded better to immunotherapy had lower DR scores. The submap analysis also showed that the low-risk group patients responded better to anti-CTLA-4 inhibitors. Therefore, we infer that patients with lower DR scores are more suitable for immunotherapy, such as anti-PD-1 and anti-CTLA-4 inhibitors. Then, we validated this result in the Braun immunotherapy cohort (CheckMate) and uncovered patients with low DR scores responded better to anti-PD1 inhibitors and had better prognosis than those with higher DR scores. The results of chemotherapy drugs IC50 showed that patients in the high-risk group were more sensitive to cisplatin, gemcitabine, and rapamycin. To sum up, the DR score could be an essential tool to help clinicians provide an individual-based treatment regime for ccRCC patients.

Nevertheless, it is essential to recognize the limitations of our research. Primarily, our retrospective study was based on the public data, and selection bias is unavoidable, which might influence the accuracy of our final results. Subsequently, external immunotherapy cohorts and prospective multicentric clinical studies are essential to authenticate the predictive ability of immunotherapy of the DR score. Finally, additional experimental evidence is necessary to uncover the potential relationship of molecular mechanisms between the DR score and disulfidptosis in RCC.

## Conclusion

In this study, we comprehensively explored the expression profiles, somatic mutation, and TME of disulfidptosis-related genes in ccRCC and established a disulfidptosis-related prognostic signature (DR score). The DR score had an excellent predictive capacity of the prognosis and was an independent risk factor. Moreover, the DR score can also predict the immunotherapy response and help clinicians provide a personalized treatment regime for ccRCC patients.

### Electronic supplementary material

Below is the link to the electronic supplementary material.

Supplementary Material 1

Supplementary Material 2

Supplementary Material 3

Supplementary Material 4

Supplementary Material 5

Supplementary Material 6

## Data Availability

The datasets supporting the conclusions of this article are included within the article and its additional files. The RNA-seq data and clinical information in this study are available in the TCGA database(https://tcga-data.nci.nih.gov/tgca/), GEO database(https://www.ncbi.nlm.nih.gov/geo/), and the E-MTAB-1980 database (https://www.ebi.ac.uk/arrayexpress/). More detailed data is available from the corresponding author on reasonable request.

## Abbreviations

RCC: renal cell carcinoma
ccRCC: clear cell RCC
ICIs: immune checkpoint inhibitors
TCGA database: The Cancer Genome Atlas database
TPM: the reanscripts per million
OS: overall survival time
DGs: disulfidptosis-related genes
DEGs: differentially expressed genes
LASSO: the least absolute shrinkage and selection operator
ssGSEA: the single-sample gene set enrichment analysis
OS-genes: OS-related DEGs
PFS: progression-free survival
CNV: copy number variation
TME: tumor microenvironment
TMB: tumor mutation burden
GO: Gene Ontology
BP: biological process
CC: cellular component
MF: molecular function
KEGG: Kyoto Encyclopedia of Genes and Genomes
GSEA: Gene Set Enrichment Analysis
LASSO: the least absolute shrinkage and selection operator
K-M survival curve: Kaplan–Meier survival curve
ROC curve: receiver operating characteristic curve
AUC: the area under the curve
PCA: principal component analysis
DEGs: differentially expressed genes
IC50: the half-maximal inhibitory concentration
TIDE: the tumor immune dysfunction and exclusion
ICI: immune checkpoint inhibitor
