# Supplementary material for: Crosstalk of disulfidptosis-related subtypes identifying a prognostic signature to improve prognosis and immunotherapy responses of clear cell renal cell carcinoma patients
Source: BMC Genomics. 2024 Apr 26;25:413. doi: 10.1186/s12864-024-10307-0 (PMC11046872; doi:10.1186/s12864-024-10307-0)
Supplement: Supplementary file 6 — Supplementary Material 6 [file 12864_2024_10307_MOESM6_ESM.docx]

**Supplementary files**

**Additional file 1:**

**Fig. S1** Protein levels of DGs and immune analysis of two DGs clusters. **A** Protein levels of DGs between ccRCC tissues and normal samples from the Human Protein Atlas database. **B-C** Differences of immune cell infiltration levels evaluated by CIBERSORT algorithm. **D** Correlation analysis of 28 immune cell subtypes infiltration levels. **E** GO analysis of DEGs in two DGs clusters.

**Fig. S2** LASSO and multivariate Cox regression. **A** The volcano plot of DEGs between two DGs clusters. **B** The LASSO coefficient distribution and 10-fold cross-validation for variable selection. **C** Forest plot shows the HR values of 8 OS-genes by multivariate Cox regression. **D** The expression profiles of 13 DGs between the low- and high-risk group in the E-MTAB-1980 cohort. **E** The distribution of DGs scores and survival status in the JAVELIN trial cohort. **F** Kaplan–Meier survival curve of two DR score group in the JAVELIN trial cohort. **G** The time-ROC curves of DR scores in the JAVELIN trial cohort.

**Fig. S3** Clinical significance of DR score. **A** Distribution of survival status, tumor stage, and T stage between the two DR score group in the Braun cohort. **B** Difference of DR scores when stratified by survival status, tumor stage, and T stage in the Braun cohort. **C** Univariate and multivariate Cox regression analyses of DR scores and clinicopathological features in the Braun cohort. **D-E** KEGG and GO analysis between two risk groups. **F** GSEA analysis showed pathways significantly enriched in the low-risk group.

**Fig. S4** Correlation analysis of DR scores and immune cell subtypes infiltration levels. **A** Differences of immune cell infiltration fractions evaluated by CIBERSORT algorithm between two risk groups. **B-C** Correlation analysis of DR scores the abundances of immune cell subtypes.

**Fig. S5** Correlation analysis of chemotherapeutic drugs IC50 and DR score.

**Fig. S6** The mRNA levels of DGs (FLNA, MYL6, NDUFS1, and SLC3A2) in three ccRCC cell lines by qRT-PCR.

**Additional file 2: Table S1** The clinicopathological information of the TCGA, E-MTAB-1980, Braun, JAVELIN trial cohorts.

**Additional file 3: Table S2** The detailed information of 13 disulfidptosis-related genes, differentially expressed genes between two DGs clusters, and OS-related differentially expressed genes.

**Additional file 4: Table S3** The immune, Stromal, ESTIMATE, and TMB scores of each patient in the TCGA cohort.

**Additional file 5: Table S4** Primers for qRT-PCR.
